# Supplementary material for: Frontotemporal dementia mutant Tau promotes aberrant Fyn nanoclustering in hippocampal dendritic spines
Source: eLife. 2019 Jun 25;8:e45040. doi: 10.7554/eLife.45040 (PMC6592683; doi:10.7554/eLife.45040)
Supplement: Supplementary file 1. [file elife-45040-supp1.docx]

**Supplementary Table S1: Summary of statistical analyses.**

| Figure | Metric | n | | Statistical test | Post-hoc correction | p value | Degree of freedom (df) and t value (t) |
| --- | --- | --- | --- | --- | --- | --- | --- |
| Fig. 1l | AUC | 31, 14 | neurons | Two-tailed Mann-Whitney U test |  | 0.0014 |  |
| Fig. 1n | Immobile fraction | 31, 14 | neurons | Two-tailed, unpaired Student's t test |  | 0.0295 | t = 2.25, df = 43 |
| Fig. 2f | AUC | 12 | neurons | Two-tailed, paired Student's t test |  | <0.0001 | t = 6.41, df = 11 |
| Fig. 2h | Immobile fraction | 12 | neurons | Two-tailed, paired Student's t test |  | 0.0028 | t = 3.84, df = 11 |
| Fig. 3d | S1 occupancy | 12 | neurons | Two-tailed, paired Student's t test |  | 0.0412 | t = 2.31, df = 11 |
|  | S2 occupancy | 12 | neurons | Two-tailed, paired Student's t test |  | 0.0157 | t = 2.85, df = 11 |
|  | S3 occupancy | 12 | neurons | Two-tailed, paired Student's t test |  | 0.0029 | t = 3.8, df = 11 |
| Fig. 4c | Average intensity | 13 | neurons | Two-tailed, unpaired Student's t test |  | <0.0001 | t = 6.41, df = 22 |
| Fig. 5f | AUC | 14, 9 | neurons | Two-tailed, unpaired Student's t test |  | 0.2787 | t=1.11, df = 21 |
| Fig. 5h | Immobile fraction | 14, 9 | neurons | Two-tailed, unpaired Student's t test |  | 0.2727 | t=1.3, df = 21 |
| Fig. 5j | AUC | 14, 9 | neurons | Two-tailed, unpaired Student's t test |  | 0.7389 | t=0.34, df = 21 |
| Fig. 5l | Immobile fraction | 14, 9 | neurons | Two-tailed, unpaired Student's t test |  | 0.7721 | t=0.29, df = 21 |
| Fig. 5n and  Fig. 7b* | Effective diffusion coefficient | 133, 79 | Spines | Kruskal-Wallis test | Dunn’s test | 0.5584 |  |
|  |  | 133, 76 | Spines | Kruskal-Wallis test | Dunn’s test | 0.8344 |  |
|  |  | 76, 124 | Spines | Kruskal-Wallis test | Dunn’s test | < 0.0001 |  |
| Fig. 5o* | Effective diffusion coefficient | 73, 43 | Shaft segments | Kruskal-Wallis test | Dunn’s test | 0.0169 |  |
|  |  | 73, 72 | Shaft segments | Kruskal-Wallis test | Dunn’s test | 0.6352 |  |
| Fig. 6d | AUC | 11, 9 | neurons | Two-tailed, unpaired Student's t test |  | 0.0014 | t = 3.78, df = 18 |
| Fig. 6f | Immobile fraction | 11, 9 | neurons | Two-tailed, unpaired Student's t test |  | 0.0014 | t = 3.76, df = 18 |
| Fig. 6h | AUC | 11, 9 | neurons | Two-tailed, unpaired Student's t test |  | 0.0245 | t = 2.45, df = 18 |
| Fig. 6j | Immobile fraction | 11, 9 | neurons | Two-tailed, unpaired Student's t test |  | 0.0373 | t = 2.25, df = 18 |
| Fig. 7c | MSS slope | 2282,  3764 | Trajectories | Kolmogorov-Smirnov D test |  | <0.0001 |  |
| Fig. 7e | S1 occupancy | 11, 9 | neurons | Two-tailed, unpaired Student's t test |  | 0.3485 | t = 0.96, df = 18 |
|  | S2 occupancy | 11, 9 | neurons | Two-tailed, unpaired Student's t test |  | 0.0049 | t = 3.2, df = 18 |
|  | S3 occupancy | 11, 9 | neurons | Two-tailed, unpaired Student's t test |  | 0.0079 | t = 2.98, df = 18 |
| Fig. 7f | Nanodomain diameter | 11, 9 | neurons | Two-tailed, unpaired Student's t test |  | 0.054 | t = 2.06, df = 18 |
| Fig. 7g | Nanodomain area | 11, 9 | neurons | Two-tailed, unpaired Student's t test |  | 0.062 | t = 1.99, df = 18 |
| Fig. 7h | # Nanodomain per spine | 11, 9 | neurons | Two-tailed, unpaired Student's t test |  | 0.0047 | t = 3.22, df = 18 |
| Fig. 8c | AUC | 8, 11 | neurons | Two-tailed Mann-Whitney U test |  | 0.9768 |  |
| Fig. 8e | Immobile fraction | 8, 11 | neurons | Two-tailed Mann-Whitney U test |  | 0.9678 |  |
| Fig. 8g | AUC | 8, 11 | neurons | Two-tailed, unpaired Student's t test with Welch’s correction |  | 0.9747 | t = 0.03, df = 13.51 |
| Fig. 8i | Immobile fraction | 8, 11 | neurons | Two-tailed, unpaired Student's t test with Welch’s correction |  | 0.7054 | t = 0.39, df = 11.6 |
| Fig. 8k | Effective diffusion coefficient | 99, 181 | Spines | Two-tailed Mann-Whitney U test |  | 0.3687 |  |
| Fig. 8l | MSS slope | 2562,  4943 | Trajectories | Kolmogorov-Smirnov D test |  | 0.4756 |  |
| Fig. 8m | S1 occupancy | 8, 11 | neurons | Two-tailed, unpaired Student's t test |  | 0.1464 | t = 1.52, df = 17 |
|  | S2 occupancy | 8, 11 | neurons | Two-tailed, unpaired Student's t test |  | 0.3416 | t = 0.98, df = 17 |
|  | S3 occupancy | 8, 11 | neurons | Two-tailed, unpaired Student's t test |  | 0.6796 | t = 0.42, df = 17 |

*p values adjusted for multiple comparisons are reported
